# From in silico to in vitro: a trip to reveal flavonoid binding on the *Rattus norvegicus* Kir6.1 ATP-sensitive inward rectifier potassium channel

**DOI:** 10.7717/peerj.4680

**Published:** 2018-05-02

**Authors:** Alfonso Trezza, Vittoria Cicaloni, Piera Porciatti, Andrea Langella, Fabio Fusi, Simona Saponara, Ottavia Spiga

**Affiliations:** 1 Department of Biotechnology, Chemistry and Pharmacy, University of Siena, Siena, Italy; 2 Toscana Life Sciences Foundation, Siena, Italy; 3 Department of Life Sciences, University of Siena, Siena, Italy

**Keywords:** Potassium channel, ATP-sensitive inward rectifier potassium channel, Binding site, Homology modeling, Patch clamp, Molecular dynamics, Molecular docking, Kir6.1, Flavonoid

## Abstract

**Background:**

ATP-sensitive inward rectifier potassium channels (Kir), are a potassium channel family involved in many physiological processes. K_ATP_ dysfunctions are observed in several diseases such as hypoglycaemia, hyperinsulinemia, Prinzmetal angina–like symptoms, cardiovascular diseases.

**Methods:**

A broader view of the K_ATP_ mechanism is needed in order to operate on their regulation, and in this work we clarify the structure of the *Rattus norvegicus* ATP-sensitive inward rectifier potassium channel 8 (Kir6.1), which has been obtained through a homology modelling procedure. Due to the medical use of flavonoids, a considerable increase in studies on their influence on human health has recently been observed, therefore our aim is to study, through computational methods, the three-dimensional (3D) conformation together with mechanism of action of Kir6.1 with three flavonoids.

**Results:**

Computational analysis by performing molecular dynamics (MD) and docking simulation on rat 3D modelled structure have been completed, in its closed and open conformation state and in complex with Quercetin, 5-Hydroxyflavone and Rutin flavonoids. Our study showed that only Quercetin and 5-Hydroxyflavone were responsible for a significant down-regulation of the Kir6.1 activity, stabilising it in a closed conformation. This hypothesis was supported by in vitro experiments demonstrating that Quercetin and 5-Hydroxyflavone were capable to inhibit K_ATP_ currents of rat tail main artery myocytes recorded by the patch-clamp technique.

**Conclusion:**

Combined methodological approaches, such as molecular modelling, docking and MD simulations of Kir6.1 channel, used to elucidate flavonoids intrinsic mechanism of action, are introduced, revealing a new potential druggable protein site.

## Background

Potassium channels are the most various ion channel family group (Perney & Kaczmarek, 1991; Luneau et al., 1991). Each category of K^+^ channel is activated by several signals and environments depending on their nature of regulation: some open in reaction to depolarisation of the plasma membrane; others in reaction to hyperpolarisation or a growth in intracellular calcium concentration; some can be regulated after the binding of a transmitter, others are regulated by GTP-binding proteins or other messengers (Schwarz et al., 1988). Inwardly rectifying potassium channels (Kir) are the main important group of two TMD potassium channels. The Kir superfamily comprises 16 members in seven different subfamilies, from the Kir1 to the Kir7 (Doupnik, Davidson & Lester, 1995; Lu, 2004) and their function is influenced by their gating features, which is attended by conformational transitions. Four Kir subunits are assembled into a tetrameric channel complex which is composed by hetero or homomeric subunits (Glaaser & Slesinger, 2015). Inwardly rectifying potassium channels is a ubiquitous potassium channel family ordered in two transmembrane domains (TMDs) which regulate several physiological processes including cellular excitability, heart rate, vascular tone, renal salt flow and insulin release (Minor et al., 1999). Physiological activity and role of Kir channels depend on regulation of the pore opening, ion flux and channel position (Meng et al., 2016). Our study was focused on *Rattus norvegicus* ATP-sensitive inward rectifier potassium channel 8 (Kir6.1) belonging to the K_ATP_ subfamily (Stephan et al., 2006). It shows four subunits with two TMDs that are linked with a P-domain (P loop), the latter representing the ion-selective domain (selectivity filter). All K^+^ channels have a core of alpha subunits, each comprising one or two copies of conserved pore loop domain (P-domain), which contains the sequence (T/SxxTxGxG) (Miller, 2000). A second ‘pore,’ known as G loop, is localised in the cytoplasmic domain (CTD), where the pore is a typical architecture of Kir channels (Li et al., 2014). Thus, three gates are distributed along the permeation way: selective filter gate, the bundle-crossing gate in transmembrane pore and G loop gate, in the cytoplasmic one. The TMD and CTD are linked by about 20 amino acids and four supporting proteins, the sulfonylurea receptors, which envelop the four subunits of K_ATP_ forming a hetero-octameric complex (Sepúlveda et al., 2015). The Kir channel open-closed mechanism depends on different conformational changes which regulate its state (Li et al., 2016; Lü et al., 2016). Small molecules regulate functions of Kir channels for instance: H^+^, Mg^2+^, Na^+^; polyamines, phosphorylation and membrane-bound phospholipids and proteins (Xie et al., 2007; Hibino et al., 2010; Li et al., 2016; Fowler et al., 2014). K_ATP_ channels act as an endogenous homeostatic transducer in response to an altered demand (Carrasco et al., 2001; Zingman et al., 2002; Miki et al., 2002; Gumina et al., 2003). In the heart, they protect against ischemia metabolic insult and contribute, as molecular mediator, to the adaptive response to stress. They regulate vascular tone, metabolic resource delivery (Lawson & Dunne, 2001; Cole & Clément-Chomienne, 2003) and are crucial in blood–glucose level setting by regulating pancreatic β-cells insulin secretion and skeletal muscle insulin-dependent glucose uptake (Aguilar-Bryan, Bryan & Nakazaki, 2001; Minami et al., 2004). Similarly, in the brain, K_ATP_ channel stimulation has a protective role against metabolic challenge (Yamada et al., 2001). Therefore, K_ATP_ channels, combined with cellular and systemic metabolism, act at various levels to guarantee metabolic health under stress challenge (Zingman et al., 2002). Potassium channel openers play a role in matching membrane electrical excitability with variations in energetic state, and also in preserving metabolic expenditure (Yamada, 2010), making these molecules cytoprotective agents under varied conditions (Coghlan, Carroll & Gopalakrishnan, 2001; Jahangir, Terzic & Shen, 2001; Campbell, Sansom & Ashcroft, 2003; Mannhold, 2004). Thus, potassium channel openers could have important benefit as myocardial protectors, antihypertensive vasodilators, bronchodilators, bladder relaxants, islet cell protectors and antiepileptics (Yokoshiki et al., 1998; Tinker, Aziz & Thomas, 2014; Villa & Combi, 2016). Li et al. (2017) and Martin et al. (2017) discovered the first structure of a Pancreatic ATP-sensitive Potassium Channel in its closed state (Kir6.2), elucidating many of its structural and regulating aspects (Martin et al., 2017; Li et al., 2017).

Several studies showed that Kir channel have the capacity to interact with different types of molecules (Mackie et al., 1995; Zhang et al., 2011; Gribble & Reimann, 2002; Miura & Miki, 2003; Kaufmann et al., 2013; Matsushita & Puro, 2006; Pattnaik & Hughes, 2009), among these flavonoids (Chiang et al., 2002; Macêdo et al., 2014; Ogata et al., 1997; Ma et al., 2014). Flavonoids are natural polyphenolic agents found in all plants (Mattila, Astola & Kumpulainen, 2000) and usually consumed in significant amounts from beverages, fruits and vegetables. Flavonoids have important health benefits: decreasing heart disease (Renaud & de Lorgeril, 1992), giving a protective role against cancers (Kandaswami et al., 1991; Hertog et al., 1995) and neurodegenerative diseases (Mandel & Youdim, 2004). Thus, flavonoids could be considered an effective source of compounds for identifying compounds with different mechanisms of action. It is commonly known that some flavonoids interact with inward rectifier potassium ion channel (Kir) and they are able to inhibit them, as genistein, an isoflavone with inhibitory activity among three members of Kir family (Kir 2.3, Kir 2.1 and Kir 3.4), resulting in beneficial effects on the cardiovascular system (Zhao et al., 2008) and beneficial epidemiological effects (Huxley & Neil, 2003). For instance, naringin and naringenin, are bioflavanoids found in grapefruit. Yow et al. (2011) explained the mechanism of their action on the potential binding site of Kir3 channels: naringin but not naringenin activates Kir3 channels. Thus, a deeper understanding about the effects of flavonoids on vascular potassium currents, in particular on Kir, is needed in order to elucidate flavonoids’ intrinsic mechanism of action and to reveal potential druggable protein sites. As reported in previous work, the flavonoid genistein is responsible for the inhibition of the most prominent flavonoids in fruits and vegetables are flavonols, and, of these, Quercetin is the most commonly taken in with human diet, for this reason we chose Quercetin to start our flavonoids–Kir channel interactions study (Ko et al., 2009).

Unfortunately, Kir6.2 has been resolved with a low resolution and no resolved K_ATP_ open structure is currently available. Despite previous works demonstrating the involvement of Kir CTD in the gating regulation and a G loop crucial involvement in the K_ATP_ channels gating process (Pegan et al., 2005; Hansen, Tao & MacKinnon, 2011; Li et al., 2015), many aspects of its structure, mechanism and modulation remain still obscure. Using a bioinformatics approach, we modelled the structure of the closed and open state of the ATP-sensitive inward rectifier potassium channel 8 and revealed an inhibition role of flavonoids against Kir6.1.

## Materials and Methods

### Homology modelling

The primary sequences of *R. norvegicus* inward rectifier K^+^ channel Kir6.1 (Q63664 Uniprot code) were acquired from Uniprot in FASTA format (The UniProt Consortium, 2017). PHYRE2 and I-TASSER server (Kelley et al., 2015; Wang et al., 2017) were used in order to achieve a protein structure prediction. Template crystal structure of the G protein-gated inward rectifier K^+^ channel GIRK2 in closed and open state with 3SYA and 3SYQ PDB code respectively was chosen for its genetic relationship and it was downloaded from RCSB Protein Data Bank (Whorton & MacKinnon, 2011). Model optimisation was completed using Ramachandran plot calculations which were computed with the PROCHECK program (Laskowski, 2003). Energy minimisation protocol was carried out on Kir6.1 three-dimensional (3D) models by using GROMACS 4.5. Root mean square deviation (RMSD) was computed using GROMACS 4.5 software package (Pronk et al., 2013).

### Molecular docking

The molecular structure of Quercetin, 5-Hydroxyflavone and Rutin (5280343, 68112, 5280805 PubChem CID respectively) were acquired through PubChem in sdf format (Kim et al., 2016). A docking simulation study of ligands against Kir6.1 channel closed state was performed by using flexible side chains protocol based on Iterated Local Search Global Optimizer Algorithm of AutoDock/VinaXB (Trott & Olson, 2010). The pdbqt format, essential for docking simulation, were generated by using Open Babel tools, adding Gasteiger charge (O’Boyle et al., 2011), whereas the pdbqt format of proteins were generated using a scripts included in the Autodock/VinaXB tools. Protein–ligands network interaction was evaluated with protein–ligand interaction profiler (PLIP) (Salentin et al., 2015). PyMOL 1.7.6.0 was used as molecular graphics system (The PyMOL Molecular Graphics System, Version 1.8; Schrödinger, LLC, New York, NY, USA). Through the use of ABS-scan tool 2 (Anand et al., 2014) an in silico alanine scanning mutagenesis was carried out. The amino acid residues involved in the binding site were computationally substituted to alanine and their interactions energy were recalculated. The obtained ΔΔ*G* values were computed by comparing them with the wild type sequence allowing the individual evaluation of each residue contribution.

### Molecular dynamics simulation

Molecular dynamics (MD) protocol was applied for closed and open state of inward rectifier K^+^ channel Kir6.1. Furthermore, we carried out a MD simulation against Kir6.1-Quercetin and 5-Hydroxyflavone complex. MD simulations of Kir6.1 channel in closed and open state were implemented using the GROMACS version 4.5.5 package with GROMOS 53A6 force field (Pronk et al., 2013). The channels were immersed in an explicit palmitoyloleoyl-phosphatidylcholine (POPC) bilayer (Li et al., 2015; Haider et al., 2007). The proteins were placed in a cubic box solvated with TIP3P type of water molecules. The systems were neutralised with Cl^−^ counter ions and a concentration of 0.1 M NaCl was added to the system. Both the proteins were energetically minimised using the steepest descent algorithm. The systems were equilibrated with NVT and NPT ensemble protocols for 100 and 500 ps respectively. The temperature of the simulation system was set to 300 K. The MD simulations were run with 2 fs time steps and they were performed for 50,000 ps (50 ns). MD simulation against Kir6.1-Quercetin and 5-Hydroxyflavone complex was equally performed using GROMACS version 4.5.5 package but differ from the previous ones in the applied force field (GROMOS96 43A1). The initial complex structures were obtained following a docking simulation. Topology and all parameters of ligands were evaluated and downloaded through PRODRG server (Schüttelkopf & van Aalten, 2004). The complexes were solvated in a cubic box containing a simple point charge as type of water molecules. The systems were neutralised and the NVT, NPT and MD protocols were applied.

### Cell isolation procedure

Smooth muscle cells were freshly isolated from the tail main artery by means of collagenase (type XI) treatment, as described by Mugnai et al. (2014). All animal care and experimental protocols conformed to the European Union Guidelines for the Care and the Use of Laboratory Animals (European Union Directive 2010/63/EU) and had been approved by the Italian Department of Health (666/2015-PR).

### Whole-cell patch clamp recordings

The conventional whole-cell patch-clamp method was employed to record Kir_ATP_ currents at room temperature (20–22 °C), as described by Liang et al. (2011). Borosilicate glass recording electrodes had a pipette resistance of 2–5 MΩ. Membrane currents, low-pass filtered at 1 kHz and digitised at 3 kHz, were recorded at a steady membrane potential (*V*_h_) of −50 mV, using a continuous gap-free acquisition protocol, by means of an Axopatch 200B patch-clamp amplifier (Molecular Devices Corporation, Sunnyvale, CA, USA). The osmolarity of the external solution (330 mosmol) and that of the internal solution (304 mosmol) were measured with an osmometer (Osmostat OM 6020; Menarini Diagnostics, Florence, Italy). Kir_ATP_ current values were corrected for leakage using 10 μM glybenclamide, which blocked Kir_ATP_ currents.

### K_ATP_ current recording

External solution contained (in mM): 25 NaCl, 140 KCl, 10 HEPES, 10 glucose, 1 MgCl_2_, 0.1 CaCl_2_ and 1 tetraethylammonium (TEA); pH was adjusted to 7.4 with NaOH. The internal solution consisted of (in mM): 140 KCl, 10 HEPES, 10 EGTA, 1 MgCl_2_, 5 glucose, 0.1 Na_2_ATP, 1 KADP and 0.1 Na_2_GTP; pH was adjusted to 7.3 with KOH. To minimise voltage-dependent K^+^ currents, K_ATP_ currents were recorded at a steady membrane potential (*V*_h_) of −50 mV using a continuous gap-free acquisition protocol. Current values were corrected for leakage using 10 μM glybenclamide, which completely blocked K_ATP_ currents.

### Chemicals

Collagenase (type XI), trypsin inhibitor, bovine serum albumin, tetraethylammonium chloride, EGTA, HEPES, taurine, pinacidil and glybenclamide were from Sigma Chimica (Milan, Italy). Stock solutions of Quercetin, 5-Hydroxyflavone, Rutin, pinacidil and glybenclamide, dissolved directly in DMSO, were stored at −20 °C and protected from light. DMSO and ethanol (below 0.1%, v/v) did not affect current amplitude.

### Statistical analysis

pClamp 9.2.1.8 software (Molecular Devices Corporation, Sunnyvale, CA, USA) and GraphPad Prism version 5.04 (GraphPad Software Inc., San Diego, CA, USA) were used to analyse the data. Data are reported as mean ± SEM; *n* is the number of cells analysed (indicated in parentheses), isolated from at least three animals. Statistical comparisons were performed by either one-way ANOVA (followed by Dunnett post-hoc test) or Student’s *t*-test for paired samples (two tailed) (GraphPad Prism version 5.04). Post-hoc tests were performed only when ANOVA found a significant value of *F* and no variance in homogeneity. In all comparisons, *P* < 0.05 was considered significant.

## Results

**Figure 1:**
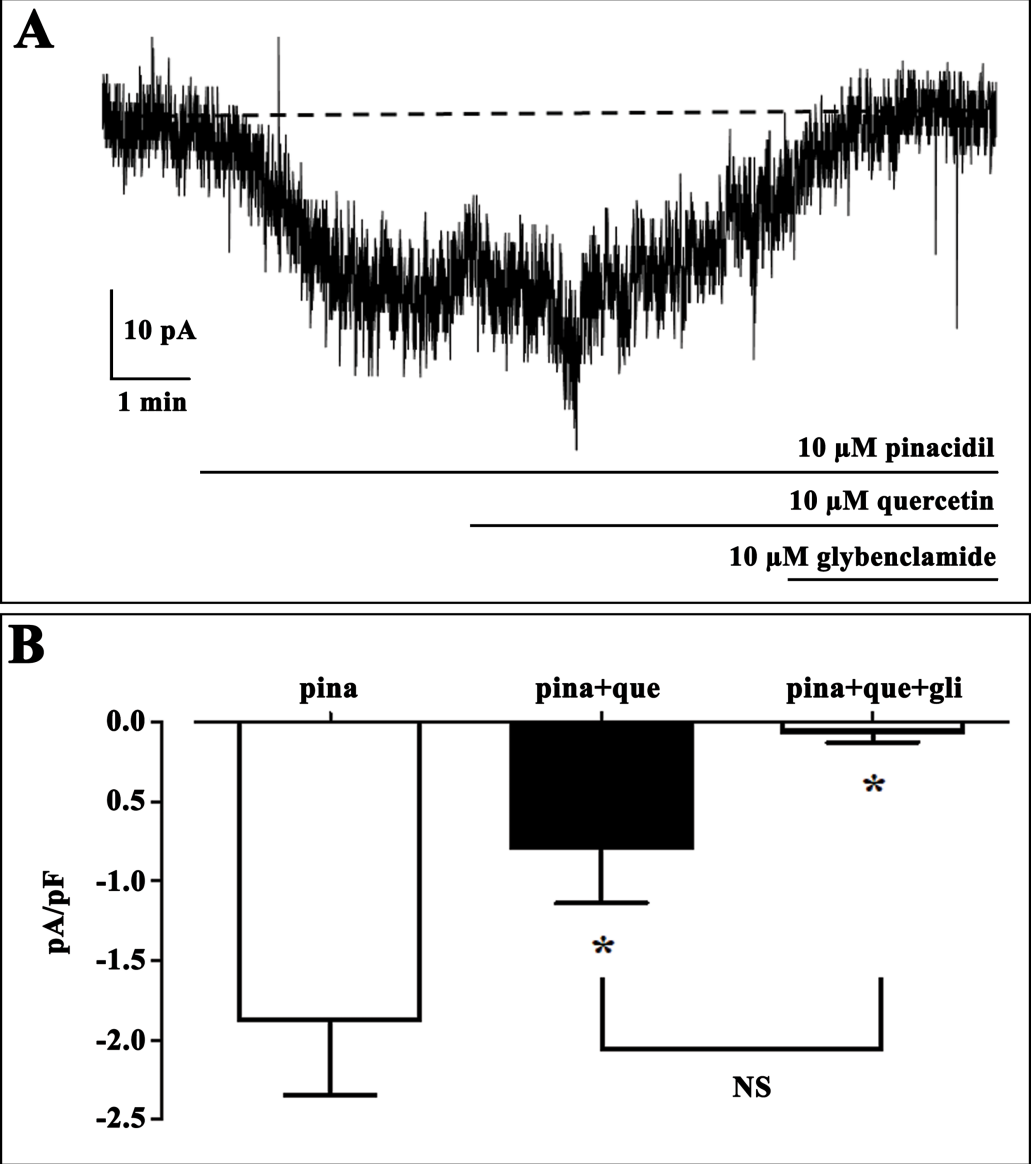
Effect of quercetin on K_ATP_ currents of isolated rat tail artery myocytes. (A) Representative whole-cell recordings of inward currents elicited by pinacidil at a *V*_h_ of −50 mV. The effect of quercetin as well as glibenclamide is shown. (B) Pinacidil (10 μM; pina) activated glibenclamide (gli)-sensitive K_ATP_ currents, which were inhibited by 10 μM quercetin (que). Columns are mean ± SEM (*n* = 6). *P < 0.05 vs. pinacidil alone, repeated measures ANOVA and Bonferroni post-test.

The capacity of Quercetin to interact with Kir6.1 channel was firstly tested with the in vitro assay. The patch clamp technique results obtained by testing Quercetin on Kir6.1 channel of *Rat norvegicus* aorta shows its inhibiting activity Fig. 1. We use computational studies to identify the potential binding pocket of this specific inhibitor and its drug ability against the Rat Kir6.1 protein.

**Figure 2:**
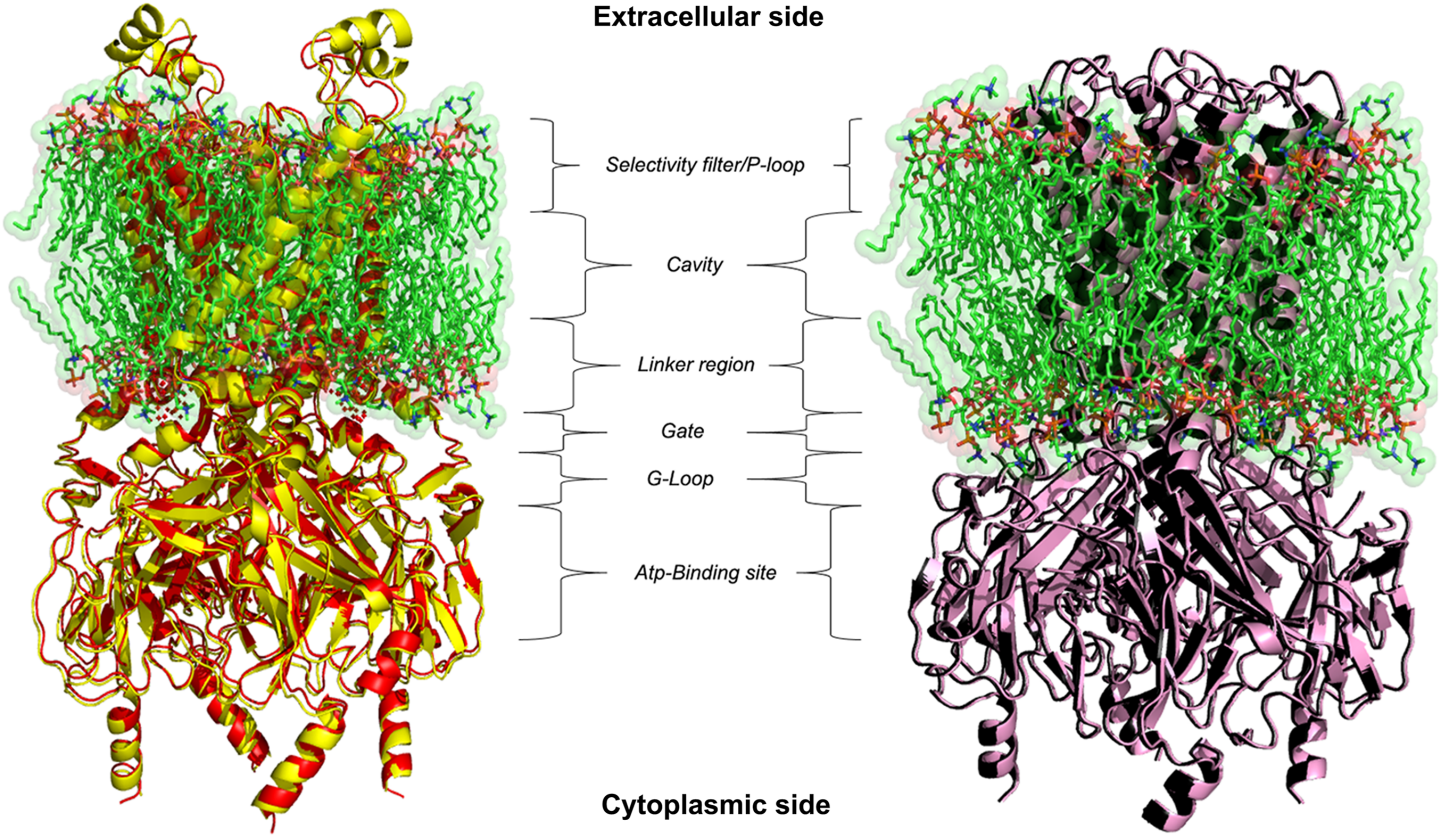
3D homology models. Ribbon representation of Kir6.1 homology modelling in closed (red) and open (black) state against Kir3.2 structure templates (crystal structures PDB entries: 3SYA yellow and 3SYQ pink) inside the cell membrane.

BLAST-Protein analysis results revealed a perfect reliable template, i.e. crystal structures of Kir6.2 from Rat and Human with a 6.3 and 5.6 Å resolutions (PDB ID: 5TWV and 5WUA) shared 90% query coverage and 70.42% identity with 0.0 *E*-value, but its refinement does not seem to be trustworthy with this low templates resolution. Attempts to optimise our model, through homology modelling, were carried out in order to achieve the 3D structure of the *R. norvegicus* Kir6.1, choosing as template the crystal structure of Kir3.2 with 2.98 Å resolution. The chosen template is member of the same Kir channel family with 50% identity and 78% conservative amino acids respectively in its closed and open states (PDB ID: 3SYA and 3SYQ). Based on alignment, two different 3D models were generated for target protein by using PHYRE2 and I-TASSER (Kelley et al., 2015; Wang et al., 2017) modelling and the missing sidechain were added and aligned from SwissPDBViewer v3.7 program (Johansson et al., 2012). Structural superimposition between Kir6.1 model and X-ray structures 3SYA and 3SYQ reported in Fig. 2, showed a high structural similarity with backbone RMSD values of 0.55 and 0.74 Å respectively. Structure validation results stated that homology modelled protein *R. norvegicus* Kir6.1 possesses reasonable 3D structure with good stereo-chemical quality of Ramachandran plot where PROCHECK analysis showed most favoured regions with 97.2% and 97.3% of the amino acid residues for closed and open state respectively. The validated structures of rat Kir6.1 were further subjected to an energy minimisation to get a reliable conformation in order to proceed for molecular docking studies. FTsite program (Ngan et al., 2012) analysis was needed in order to obtain reasonable size binding-pockets of the protein models which could be involved in interactions with Quercetin. FTsite was performed on both of states, however, no reliable volume binding-site, needed by Quercetin molecule, was detected in the open one.

The most interesting observed pocket is a conservative site, consisting in physical gates formed by cytoplasmic (G loop) region (Hibino et al., 2010), necessary for the regulation of the inward potassium current that obviously depends on the state of the gate (Lü et al., 2016). The G loop has a crucial role with their amino acid composition in the regulation of K_ATP_ gating kinetics, different previous works shown how mutations of the amino acid of this region are responsible of protein inactivation (Shimomura et al., 2009; Li et al., 2016; Nishida et al., 2007; Pegan et al., 2006; Hattersley & Ashcroft, 2005; Proks et al., 2005).

**Figure 3:**
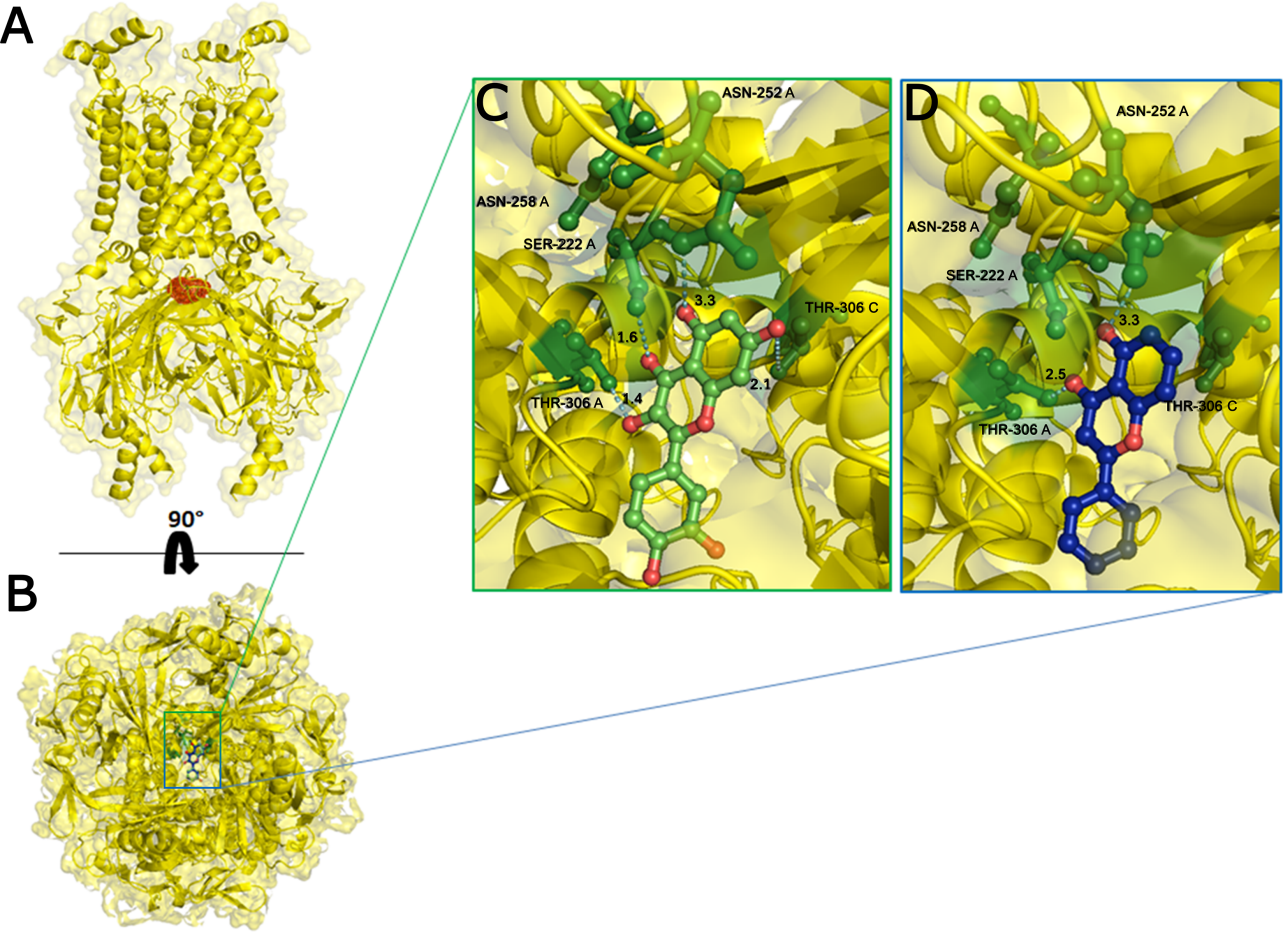
Docking results. The side view of FTsite predicted Kir6.1 binding region (red mesh) (A). The top view from the cytoplasmic side of binding region with flavonoid ligands (B). Representation of binding pocket complexed with Quercetin in green (C) and 5-Hydroxyflavone in blue (D), and binding interaction residues (green ball and sticks) after 50 ns of MD simulations.

**Figure 4:**
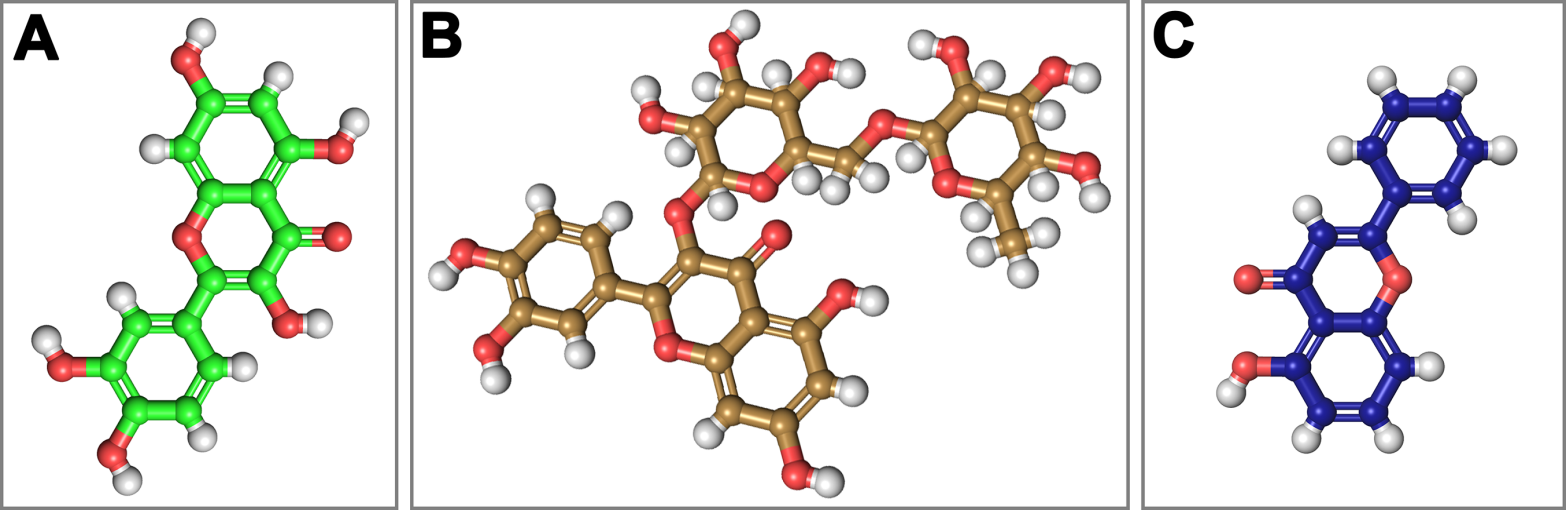
Structural 2D representations of flavonoids in ball and stick. (A) Quercetin, (B) Rutin, (C) 5-Hydroxyflavone.

**Figure 5:**
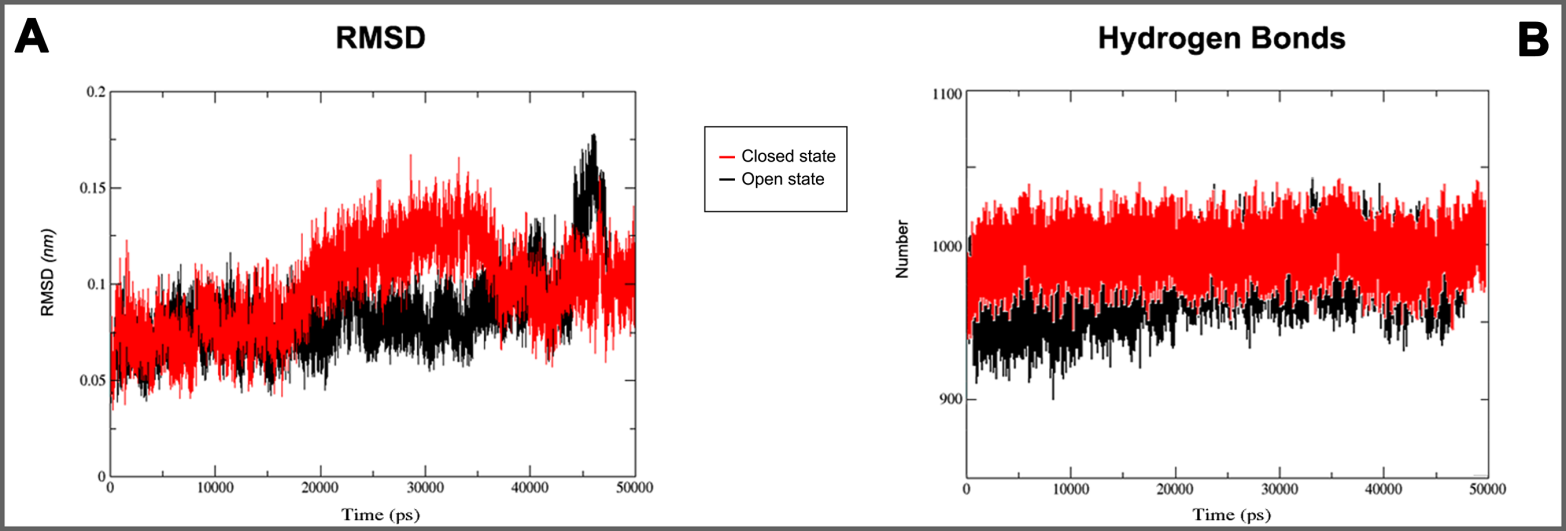
3D models molecular dynamics. (A) GROMACS MD simulation plot profiles of 50 ns backbone RMSD and (B) time evolution of hydrogen bonds. In red closed and in black open Kir6.1 conformation.

**Figure 6:**
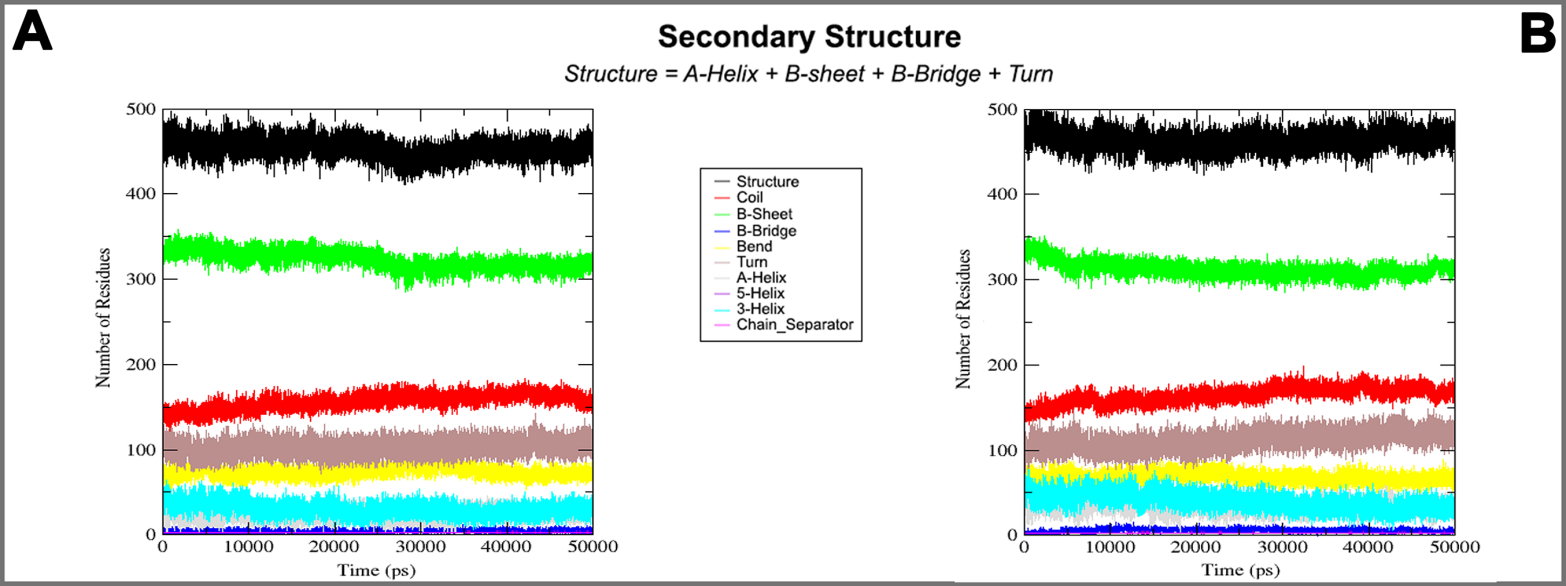
Secondary structure during MD simulations. In the plots analysis of secondary structure elements of closed (A) and open (B) conformation during MD simulations.

**Figure 7:**
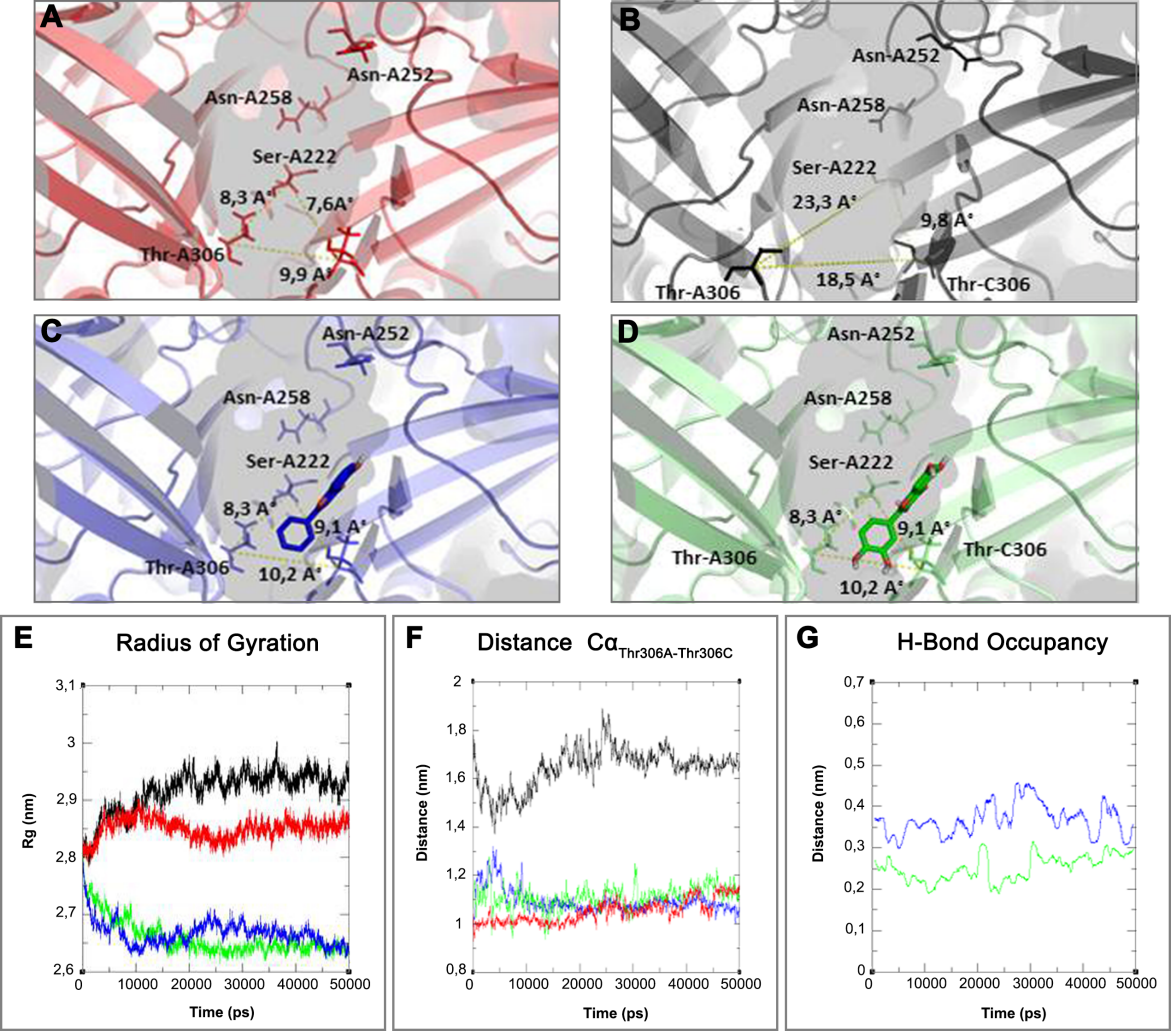
3D structure and MD simulations comparison. On the top binding pocket of Kir6.1 and the residues that comprise it in the open (A) and closed (B) state. It is mainly composed by the amino acids of G loop (thin sticks) with different positions and dimensions of the binding pocket. In (A–D) are the same binding pockets after molecular dynamics simulation (C) of 5-Hydroxyflavone (blue bold sticks) and (D) Quercetin (green bold sticks) complexes obtained from molecular docking analysis. The pocket’s dimensions are highlighted with dotted yellow lines. In (E–G) three plots for the deviations time evolution, with black line representing open state, red line closed state, blue line complexed with 5-Hydroxyflavone and green line complexed with Quercetin, i.e. (E) Radius of gyration (*R*_g_) representing the protein stability against the axial force; (F) distances time evolution between Cα atoms of residues Thr-A306 and Thr-C306, selected as reference residues of binding pocket of four MD simulations; and (G) distance profile of hydrogen bonds network during simulation between Quercetin green and 5-Hydroxyflavone, the hydrogen bonds network of Quercetin is included in a range of 0.2–0.3 nm, while, the 5-Hydroxyflavone shows a range of 0.3–0.45 nm. Such evidence shows us the greatest strength and stability of the hydrogen bonds network of the Quercetin in comparison with 5-Hydroxyflavone.

On the basis of above mentioned studies, we focused our analysis on the site involving G loop region. The virtual docking, based on virtual screening using AutoDock/VinaXB (Trott & Olson, 2010), was firstly carried out with the ligand Quercetin, in order to confirm the reliability of G loop as pocket and interaction site. The ligand–protein interactions were analysed through PLIP bioinformatics tool (Salentin et al., 2015). The data reported in Fig. 3 show that Quercetin formed hydrogen bonds with the amino-groups of Asn-258 and Asn-252 of the chain A, with the hydroxyl group of Ser-222 of the chain A and with the hydroxyl group of the Thr-306 of the chains A and C. The hydrophobic interactions took place between the hydrophobic portion of Quercetin and the hydrophobic-sensing Ile-221 and Val-299 of the chain A. All these interactions take place near the G loop residues and could stabilise the K_ATP_ channel in a closed state (Li et al., 2016). The capacity of Quercetin to inhibit Kir6.1 protein is in agreement with already published data (Chiang et al., 2002; Xu et al., 2015; Kaufmann et al., 2013; Ogata et al., 1997; Matsushita & Puro, 2006; Pattnaik & Hughes, 2009; Ma et al., 2014), thus to validate the hypothesis hydrogen bonds network holds a crucial role in the complex stabilisation, we chose two analogue molecules 5-Hydroxyflavone (different from Quercetin for a reduced number of OH groups) and Rutin (characterised by a bulky glycoside group not present in the other ligands), structures reported in Fig. 4, and their affinity was also tested with a docking simulation in G loop region. All ligands were flexible and free to bind in the proposed binding site. Docking simulations were determined for each ligand with 10 exhaustiveness as default parameter, and the active site grid dimensions were set at *X* = 54.41 Å, *Y* = 30.78 Å and *Z* = 79.94 Å. In the docking simulation, the protein was maintained unflexible whereas the ligands and the pocket amino acids were flexible, with docking score output results representing the apparent Gibbs free energy of binding (Δ*G*_app_). Only two studied compounds, Quercetin and 5-Hydroxyflavone, manifested the capability to fit inside the pocket and to have a good binding affinity to the channel, presenting −8.1 and −6.7 kcal/mol Δ*G* values binding energies. In contrast, Rutin, despite the active site grid dimension would permit to allocate it, was not able to bind the binding site, possibly because of steric hindrance due to the presence of the disaccharide Rutinose. The negative values of predicted Δ*G*_app_ indicated that the two molecules bind to the pocket spontaneously suggesting their potential channel inhibitory binding activities. The two compounds occupied the same cavity with few amino acid changes contribution due to their conformational discrepancy and angle rotation. The variance in Δ*G* values and binding pose inside the pocket may be attributed to the differences in position of the functional groups in the two compounds. The docking results of the compounds analysed by PLIP tool (Salentin et al., 2015), giving an interaction diagram and a table of interaction data, were compared in Fig. 3. In both compounds the binding was dominated by hydrophobic interactions and hydrogen bonds, nevertheless as previously supposed, we observed a remarkable difference in their binding affinities. The explanation to this behaviour is likely due to the different hydroxyl groups present in the molecule. The Quercetin presents five hydroxyl groups whereas 5-Hydroxyflavone presents only one hydroxyl group, this difference would seem to be crucial on their mechanism of action, because Quercetin was able to form up to five hydrogen bonds, while 5-Hydroxyflavone could form only two (Fig. 3). We propose that the hydrogen bonds network, established with G loop residues, can be essential to determine inhibitor activity of different ligands. It is likely a hydrogen bond network stabilises the Kir6.1 in the closed state, likewise the presence of ligands in proximity of the pore channel would decrease solvent-accessible surface area, blocking the potassium flow (Schüttelkopf & van Aalten, 2004). Furthermore, we did not observe any Rutin docked to Kir6.1, we explain this on the basis that the binding site is not able to accommodate Rutin, given the presence of the bulky disaccharide Rutinose. After that, MD simulations were performed to evaluate the stability of the predicted 3D structure of the *R. norvegicus* Kir6.1, and its complex with Quercetin and 5-Hydroxyflavone with detailed interactions shown in Fig. 3. Often, the Homology model with further MD simulations in a entirely hydrated lipid bilayer is suitable for identifying the significant structural and dynamical data before a high-resolution experimental structure becomes available. Thus, the inherent dynamics and structural stability of TM and CTDs were investigated. The initially applied harmonic restraints on the protein backbone were gradually released during the course of the second 1 ns simulation followed by additional 50 ns MD simulations without any restraints (Ismail & Jusoh, 2016; Haider et al., 2007; Lü et al., 2016), specifying that the topology of the channel was maintained under the condition of no restraints (Figs. 5 and 6). The plot profiles suggest that the complexes with Quercetin and 5-Hydroxyflavone are able to decrease the radius of gyration in comparison with the unbound structure, Fig. 7. Additionally, we estimated the distance between key amino acids, considering C_α_ of Thr-A306 and Thr-C306 in Kir6.1 closed, open and complex states (Fig. 7). The analysis showed that the distance was bigger in the open than in the closed and complex state, interestingly, we can observe the similar profile of closed and complex state, indicating the ability of ligands to stabilise the channel in a ‘closed-like’ conformation. In addition, the radius of gyration (*R*_g_) was calculated in order to verify the compactness of the protein in absence or presence of ligands, as reported in Fig. 7. The different values of the *R*_g_ for Kir6.1 in the closed and complex state, might be due to different interactions between Quercetin and 5-Hydroxyflavone against Kir6.1 binding site, which would compact the structure.

**Figure 8:**
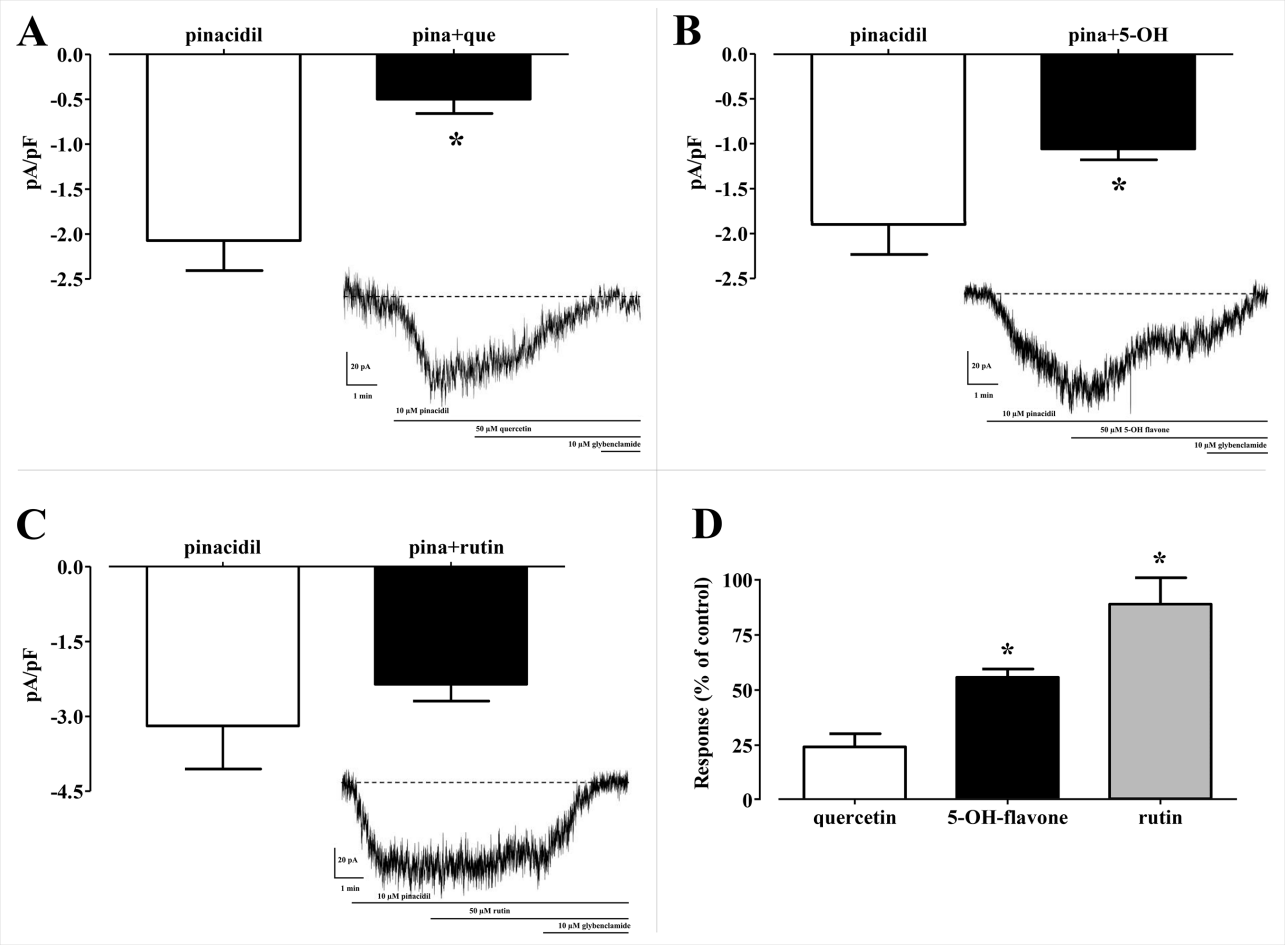
Patch clamp assay on flavonoids. Effect of various flavonoids on K_ATP_ currents of rat tail artery myocytes. (A–C) Pinacidil (10 μM) activated glibenclamide-sensitive K_ATP_ currents, which were inhibited by (A) Quercetin (que, 50 μM) and (B) 5-Hydroxyflavone (5-OH, 50 μM), but not by (C) Rutin (50 μM). *P < 0.05 vs. pinacidil alone, Student’s *t*-test for paired samples. Insets: representative recordings of inward currents elicited by pinacidil at a *V*_h_ of −50 mV. The effect of quercetin, 5-hydroxyflavone and rutin as well as glibenclamide is shown. (D) Residual K_ATP_ current measured in myocytes challenged with the three flavonoids and calculated from (A–C). Columns are mean ± SEM (*n* = 5–9). *P < 0.05 vs. 50 μM quercetin, one-way ANOVA followed by Dunnet post-hoc test.

In silico analysis of the effect of Quercetin, 5-Hydroxyflavone and Rutin on K_ATP_ channels was assessed in vitro in single myocytes, freshly isolated from the rat tail main artery, by using the conventional whole cell patch-clamp configuration. To limit activation of voltage-dependent K^+^ channels and large conductance Ca^2+^-activated K^+^ channels, K_ATP_ currents were elicited at a *V*_h_ of −50 mV in presence of 0.1 mM ATP and 1 mM ADP in the pipette solution and 1 mM TEA in the external solution. When myocytes were challenged with the K_ATP_ channel opener pinacidil (10 μM), an inward current activated (−1.62 ± 0.17 pA/pF, *n* = 5). This was significantly antagonised by the K_ATP_ channel inhibitor glibenclamide (10 μM; −0.09 ± 0.02 pA/pF, *n* = 5; *P* = 0.0005). As shown in Figs. 8A–8C, Quercetin, 5-Hydroxyflavone but not Rutin significantly inhibited the glibenclamide-sensitive currents recorded in the presence of pinacidil. Their inhibitory efficacy, however, was strikingly different (Fig. 8D), Quercetin being the most effective compound. In fact, when the concentration of Quercetin was reduced to 10 μM, current inhibition was still higher than that exerted by a fivefold greater concentration of 5-Hydroxyflavone.

**Figure 9:**
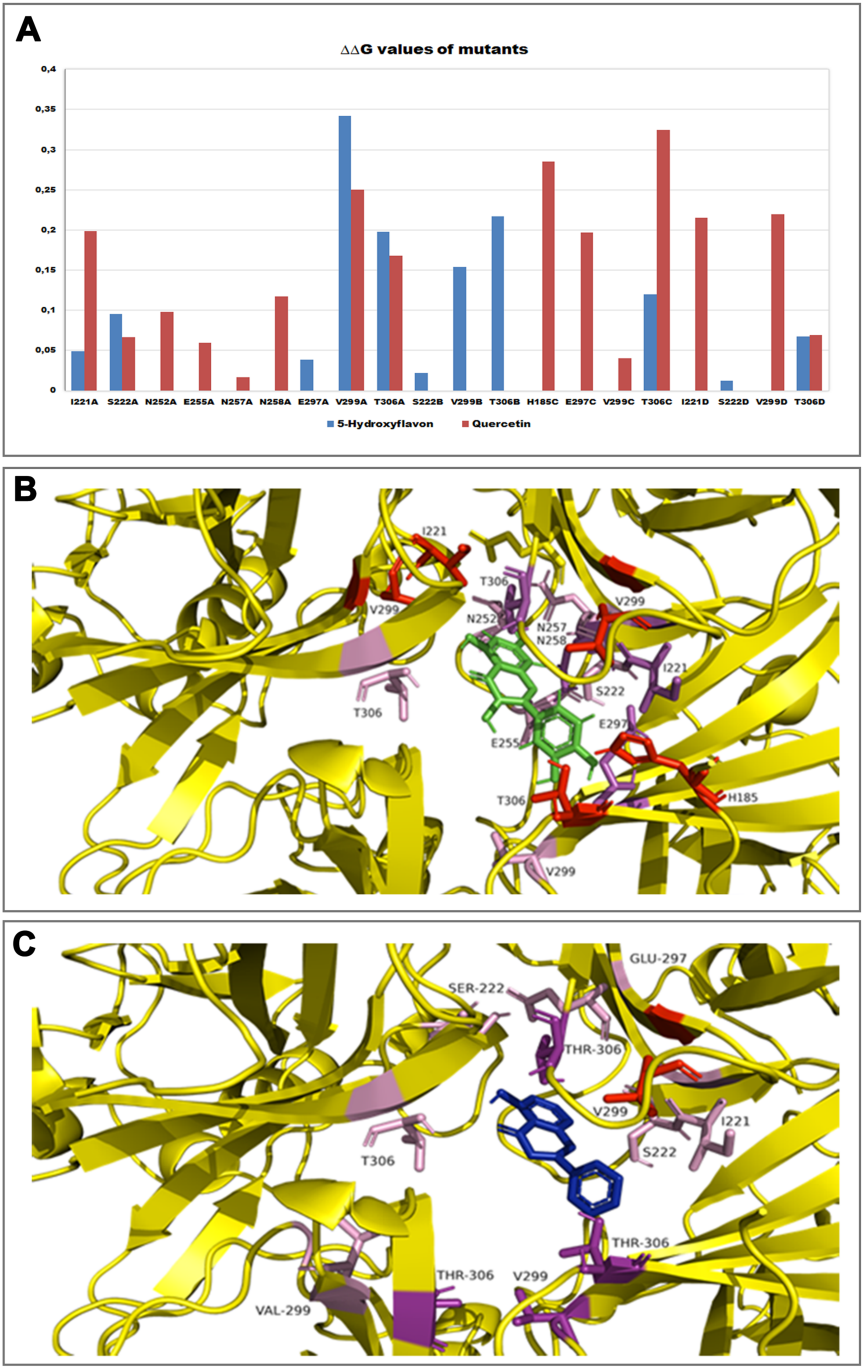
In silico alanine scanning mutagenesis. (A) ABS‐scan energy plot. ΔΔ*G*_app_ values recorded after alanine mutation of single residues involved in the binding of Quercetin and 5-Hydroxyflavone. Amino acid residues are listed in rank order according to their contribution in the complex with Quercetin (red) and 5-Hydroxyflavone (blue) (ΔΔ*G*_app_ values). (B) The residues of the binding pocket with Quercetin (green) and (C) 5-Hydroxyflavone (Blue) were shown in stick and coloured on the basis of their energetic contribution, as ΔΔ*G* terms (in pink: 0–0.10ΔΔ*G* Kcal/mol, in purple: 0.10–0.25ΔΔ*G* Kcal/mol, in red: 0.25–0.30ΔΔ*G* Kcal/mol).

Furthermore, in order to evaluate the energetic contribution of each residue involved in the interaction with the Quercetin and 5-Hydroxyflavone, we carried out an alanine-scanning simulation (Fig. 9). In Fig. 9A we reported the single contribution of residues in ΔΔ*G* terms, while in Figs. 9B and 9C was shown the docked pose of ligands inside the binding pocket. Is very interesting to note as the Quercetin (red tower) is able to bind to a major number of residues and with an higher ΔΔ*G* value in comparison with the 5-Hydroxyflavone (blue tower), these difference could be very significant about a different activity of ligands against the Kir6.1, indicating a greater inhibitory effect of the Quercetin.

## Discussion

ATP-sensitive inward rectifier potassium channel 6.1 is a potassium channel involved in many biological processes and their dysfunctions have been underlined in different pathologies. An accurate assessment of its mechanism and the identification of its potential inhibitor-binding site could be very useful for clarifying many features of Kir6.1. On the basis of knowledge that some flavonoids interact with inward rectifier potassium ion channel and their beneficial effects on the cardiovascular system we initially select Quercetin as potential candidate to identify the potential binding pocket and to understand the mechanism of action of potential inhibitors. A patch clamp technique was firstly carried out to test Quercetin effects on Kir6.1 (Fig. 1). Thus, a 3D study of this interaction was necessary to identify its mechanism of action.

Homology modelling was carried out in order to obtain a structure of Ki6.1 both in closed and open state, a protein phylogenetically correlated to the Kir6.1 was chosen as template, the crystal structure of the Kir3.2 in closed and open state (Fig. 2). The derived Kir6.1 model has been used to explore the binding mode of some flavonoids. We focused our attention on Quercetin, 5-Hydroxyflavone and Rutin (Fig. 3), in order to propose a potential consensus binding site of Kir6.1 and a flavonoid mechanism of action inside the binding pocket. Through bioinformatics approaches and previous works (Hibino et al., 2010), we identified the G loop as potential binding site for ligands (Lü et al., 2016; Shimomura et al., 2009; Li et al., 2016; Nishida et al., 2007; Pegan et al., 2006; Hattersley & Ashcroft, 2005; Proks et al., 2005). Docking simulation and different MD simulation protocols were accomplished for explaining how the flavonoids influenced the gating process of the channel. From our results, we observed that only two flavonoids, Quercetin and 5-Hydroxyflavone, were superimposed in the G loop region showing a high apparent affinity, while Rutin, possessing a bulky glycoside group (not present in Quercetin and 5-Hydroxyflavone) was not able to bind in same region (Fig. 3). In addition, the hydrogen bonds network of flavonoids in the binding site seems to be crucial for their mechanism of action. The presence of more hydroxyl groups on Quercetin could explain its greater inhibitory effect than 5-Hydroxyflavone; moreover, we showed that the ligands were able to stabilise the Kir6.1 in the closed conformation. The obtained results permitted us to classify binding site forming-residues as attachments for the inhibitor recognition process; furthermore, the interactions with the G loop pocket Ser-A222, Asn-A252, Asn-A258, Thr-A306, Thr-C306 seem to be advantageous for targeting Kir6.1 selectivity. In line with docking studies, Quercetin better occupies the binding region compared to 5-Hydroxyflavone, it performs an extensive network of hydrogen bonds due its hydroxyl groups. Some of these bonds are not very stable, but in any case provide good anchoring points for the inhibitor inside the pocket and justify selectivity and inhibitory affinity over the other two molecules, in agreement with experimental data (Fig. 8). Accordingly, the in silico data were nicely supported by the in vitro analysis of flavonoid activity towards vascular K_ATP_ channel current. Quercetin, being characterised by five OH substituents on the flavonoid scaffold, inhibited the current by about 75%. This observation is in agreement with the inhibitory effect of Quercetin on K_ATP_ channel current of INS-1 cell recently described (Kittl et al., 2016), though in insulinoma cells inhibition was transient and faded over 1 min of exposure to the drug. While 5-Hydroxyflavone showed an inhibitory effect on K_ATP_ channel current, its efficacy was lower than that of Quercetin, well correlating to the reduced number of OH groups of the molecule. Finally, Rutin, though possessing the same hydroxylation pattern of the parent compound Quercetin, was almost inactive as a K_ATP_ channel blocker. The bulky structure of the flavonoid, originating from its glycoside group, might account for its ineffectiveness towards the channel. This is in line with what previously observed on Ca_V_1.2 channel current of rat tail artery myocytes, where Quercetin proved to be a stimulator, Rutin was ineffective and 5-Hydroxyflavone was classified as a weak inhibitor of the current (Saponara et al., 2011). The evidence that their activity varied considerably, following only minor modifications in the molecular structure, further strengthens the theory that vascular channels are targets for flavonoids structurally related to Quercetin (Fusi et al., 2017). In vitro results presented in this work confirmed our computational study, showing that 5-Hydroxyflavone had a lower inhibitory activity than Quercetin of 30%, while no inhibitory effect was observed for Rutin (see Fig. 9), such evidence further confirmed our hypothesis about the existence of a flavonoid binding site on the channel protein. In agreement with these data, the in silico alanine scanning mutagenesis, Fig. 9 showed that ΔΔ*G* profile shared by Quercetin is quite different from 5-Hydroxyflavone, supporting the hypothesis that they docked in the pocket engaging different interaction networks and corroborating results obtained in vitro. The electrophysiological data, obtained under the conventional whole-cell configuration (i.e. in myocytes whose cytoplasm was subjected to extensive dialysis) neither prove a direct interaction with nor preclude an indirect effect on the channel protein. Further experiments (e.g. under the excised patch configuration) are necessary to clarify this issue.

## Conclusion

This manuscript was aimed at investigating an as yet unexplored field such as that of the potential effect of Quercetin on vascular K_ATP_ channel. A feasible mechanism of action, responsible for the current inhibition observed in vitro, was hypothesised by analysing two structurally related compounds, namely 5-Hydroxyflavone and Rutin. We believe that the good correlation found between the in silico and the in vitro results will be interesting and will stimulate further research in the field. In fact, this feature may indeed apply to other flavonoids or even other polyphenols abundantly consumed with our daily diet. Obviously, many other experiments need to be undertaken in order to define the precise mechanism of action in order to improve the drug design process of K_ATP_ inhibitors and also in using mutated Kir6.1 channels expressed in heterologous systems.

## Supplemental Information

10.7717/peerj.4680/supp-1 Supplemental Information 1 Kir61 closed structure.

10.7717/peerj.4680/supp-2 Supplemental Information 2 Kir61 open structure.

10.7717/peerj.4680/supp-3 Supplemental Information 3 Supplementary data. Raw data.

## Additional Information and Declarations

### Data Availability

The following information was supplied regarding data availability: The raw data have been provided as a Supplemental File.
