# Supplementary material for: From in silico to in vitro: a trip to reveal flavonoid binding on the Rattus norvegicus Kir6.1 ATP-sensitive inward rectifier potassium channel
Source: PeerJ. 2018 May 2;6:e4680. doi: 10.7717/peerj.4680 (PMC5936070; doi:10.7717/peerj.4680)
Supplement: Supplemental Information 3 — Raw data. [file peerj-06-4680-s003.docx]

| **Compounds** | **Hydrogen bonds** | **Hydrophobic interactions** | **ΔG_b_ (Kcal/mol)** |
| --- | --- | --- | --- |
| **Quercetin** | **Ser-A222, Asn-A252, Asn-A258, Thr-A306, Thr-C306** | **Ile-A221, Val-A299** | **- 8.1** |
| **5-Hydroxyflavone** | **Thr-C306, Asn-A252** | **Ile-A221, Val-A299** | **-6.7** |
| **Rutin** | **n.e.d*** | **n.e.d*** | **n.e.d*** |

Table 1

The stereochemical quality of the final structures (i.e. the distribution of phy and psi angles) were assessed by means of the PROCHECK program [Laskowski RA et al. 1993]. With this test, no severely disallowed atomic contacts were detected, suggesting essentially good stereochemistry, with 88.2-88.5 % and 98.8 % of the amino acids residues in the most favoured and additionally allowed regions, respectively and with 2- 1.9 % and 0.8 % residues in generously allowed and disallowed regions of the Ramachandran plot

Ramachandran plot of closed state model.

 Ramachandran plot of open state model.


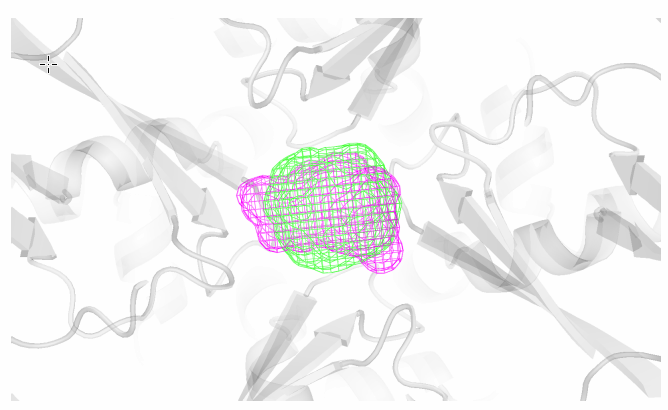


The G loop area: the open state in magenta, the closed state in green
